# Nationwide epidemiological survey of Leber hereditary optic neuropathy in Japan

**DOI:** 10.1016/j.je.2017.02.001

**Published:** 2017-04-06

**Authors:** Kaori Ueda, Yuki Morizane, Fumio Shiraga, Keigo Shikishima, Hitoshi Ishikawa, Masato Wakakura, Makoto Nakamura

**Affiliations:** a Division of Ophthalmology, Department of Surgery, Kobe University Graduate School of Medicine, Kobe, Japan; b Department of Ophthalmology, Okayama University Graduate School of Medicine, Okayama, Japan; c Department of Ophthalmology, The Jikei University School of Medicine, Tokyo, Japan; d Department of Orthoptics and Visual Science, School of Allied Health Sciences, Kitasato University, Sagamihara, Kanagawa, Japan; e Inouye Eye Hospital, Tokyo, Japan

**Keywords:** Annual incidence, Gender proportion, Leber hereditary optic neuropathy, Mitochondrial DNA, Penetrance

## Abstract

**Background:**

Leber hereditary optic neuropathy (LHON) is a maternally inherited optic neuropathy that leads to central loss of vision, predominantly in young males. Most LHON cases have one of three primary point mutations in mitochondrial DNA (mtDNA). The annual incidence and prevalence of LHON in Japan are not known. Thus, we estimated the annual incidence of molecularly confirmed LHON in Japan during 2014.

**Methods:**

Sequential questionnaires were sent to 1397 facilities, which included all of the university hospitals in Japan, and they were certified by either the Japanese Ophthalmological Society or the Japanese Neuro-Ophthalmological Society. We calculated the incidence number (*I*_*r*_) as the number of patients who developed LHON in 2014 and its 95% confidence interval.

**Results:**

We received 861 responses to the first questionnaire, where 49 facilities reported 72 cases (67 were male and 5 were female) of newly developed LHON during 2014. *I*_*r*_ was calculated as 117, and the 95% confidence interval ranged from 81 to 153. For the second questionnaire, responses were received from 30 facilities, where the median age at onset was 38 years for males and 30 years for females, and 86.5% of cases possessed the mtDNA ND4/G11778A mutation.

**Conclusion:**

Approximately 120 cases of newly developed LHON were reported during 2014 in Japan, and 93.2% were males.

## Highlights

• The incidence of Leber hereditary optic neuropathy (LHON) is unknown in Japan.
• A nationwide questionnaire survey has been done to estimate its annual incidence.
• About 120 cases of molecularly confirmed incident LHON were estimated in 2014.

## Introduction

Leber hereditary optic neuropathy (LHON) is a maternally inherited optic neuropathy characterized by acute or subacute, bilateral, painless central vision loss and eventual optic atrophy.^1^, ^2^, ^3^ LHON was the first disease to be identified as a mitochondrial DNA (mtDNA) mutation-associated human disorder. Over 95% of patients with LHON have one of the three primary point mutations in their mtDNA genes. These point mutations affect genes that encode subunits of the oxidative phosphorylation enzyme complex (ND), ND1/G3460A, ND4/G11778A, and ND6/T14484C, which are referred to as the “primary” mutations.^1^, ^2^, ^3^, ^4^ These mtDNA mutations are thought to disrupt electron transfer and increase oxidative stress, leading to apoptotic death of the retinal ganglion cells, the axons of which form the optic nerves.^1^, ^2^, ^3^, ^4^, ^5^, ^6^

Patients with LHON experience a marked reduction in visual acuity and deep central scotoma. Unlike patients with optic neuritis, those with LHON exhibit no abnormal findings on performing gadolinium-enhanced magnetic resonance imaging of the optic nerve during the acute phase of the disease, and they do not respond to steroid therapy.^1^, ^7^

LHON predominantly affects males, in whom onset occurs at adolescence or later, and it usually affects one eye initially and the other subsequently, with a mean interval of 2 months between onset in both eyes.^1^, ^7^ Up to 50% of males and 25% of females who are related through matrilineal descent to individuals with a LHON pedigree manifest symptoms. Most patients with LHON suffer from permanent vision loss, but a small fraction of patients experience spontaneous recovery of vision, even long after the onset of the disease.^1^, ^2^, ^3^, ^7^ Unlike other mitochondrial diseases, most patients with LHON do not manifest neurological symptoms other than bilateral optic neuropathy, although subclinical cardiac or skeletal muscle abnormalities have been reported.^1^, ^7^ Mutations in mtDNA alone cannot entirely account for the unique features of LHON. Therefore, additional genetic, epigenetic, or environmental factors probably influence LHON expression among pedigrees that harbor the mtDNA mutations.^8^

As found with other types of mitochondrial diseases, the prevalence and incidence of LHON have been difficult to determine because this disease is rare and burdensome to diagnose. Previous epidemiological studies have reported that LHON cases where the patients have one of the primary mtDNA mutations have a minimum point prevalence of 1 in 31,000 in the Northern United Kingdom, 1 in 39,000 in the Netherlands, 1 in 48,000 in Finland, 1 in 54,000 in Denmark, and 1 in 113,300 in Australia.^4^, ^9^, ^10^, ^11^ However, a recent study reported a minimum point prevalence of 1 in 526,000 in the Serbian population, demonstrating a possible difference in the prevalence of LHON among diverse ethnicities.^12^

Two previous epidemiological studies of LHON have been conducted in Japan using relatively large sample sizes. In 1973, Imachi et al^13^ evaluated the inheritance patterns and clinical features of 38 LHON pedigrees and 63 suspected pedigrees based on a family register and detailed clinical examinations. However, their study was conducted at a single institute and during the pre-molecular biology era. Therefore, the diagnosis of LHON may have been inaccurate, and the study may have included cases of optic neuropathy that were unrelated to LHON. Approximately 20 years later, Hotta et al^14^ conducted a survey by sending a questionnaire to 86 university hospitals and reported the clinical features of 89 patients (79 pedigrees) with the ND4/G11778A mutation from 64 institutes that responded to the questionnaire. The questionnaire was not sent to facilities other than universities, and the subjects comprised both newly developed and long-standing cases. The inclusion of the long-standing cases was thought to have led to a biased underestimate because of the dropout of a non-negligible number of patients during follow-up. In addition, patients with the other two primary mtDNA mutations were excluded from the study. Thus, these findings could not estimate the incidence or prevalence of LHON because of their incomplete study designs and selection bias. However, they provided the important insight that female penetrance might have decreased through generations, such that the proportion of males in Japanese LHON cases was reported to be 68% in 1973 but 92% in 1995.^13^, ^14^ Further epidemiological studies could help to understand the factors that trigger and regulate the symptoms of LHON in mtDNA-mutated individuals, which may facilitate the development of an effective treatment in the future.

Thus, in the current study, we estimated the annual incidence of LHON during 2014 based on a nationwide questionnaire survey as a first step toward assessing the number of patients with LHON in Japan.

## Materials and methods

### Enrollment and exclusion criteria for subjects

In this study, we used sequential questionnaires to estimate the incidence number (*I*_*r*_) as the number of patients with newly developed LHON during 2014. This study design was approved by the Ethics Committee of Kobe University Graduate School of Medicine (article No. 1711).

The diagnosis of LHON was based on the designated criteria for LHON established by the Research Committee on the Epidemiology of Intractable Diseases of Retinochoroidal and Optic Nerve Atrophy in conjunction with the Japanese Neuro-ophthalmological Society, and authorized by the Ministry of Health, Labour and Welfare, as listed in Table 1.^7^ According to the designated criteria, the diagnosis of LHON was classified into the following three categories: *definite*, *probable*, and *possible* LHON cases. In this study, we enrolled patients with LHON who had one of the three primary mtDNA mutations. Therefore, cases of *definite* and *probable* LHON were included in our study, whereas those of *possible* LHON were not considered eligible. In addition, to be eligible for the current survey, a patient had to have a recent onset of LHON and been diagnosed in the year 2014.

**Table 1:** Designated criteria of Leber hereditary optic neuropathy.^7^

I. Major symptoms ① Acute or subacute, bilateral (simultaneous or sequential), painless visual acuity reduction, and central scotoma ② At least one of the following fundus abnormalities in the acute phase: 1) redness or swelling of the optic disc 2) telangiectasia around the optic disc 3) swelling of juxtapapillary retinal nerve fibers 4) optic disc hemorrhage ③ Optic atrophy in the chronic phase
II. Ancillary testing a) Presence of mtDNA mutations undisputedly associated with LHON b) Absence of retrobulbar optic nerve abnormalities identified using computed tomography/magnetic resonance imaging in the acute phase c) Absence of dye leakage from the optic disc on fluorescein angiogram
III. Diagnosis of LHON *Definite*: A case fulfilled with the major symptoms of both ① and ② or both ① and ③ and the ancillary testing results comparable for both a) and b). *Probable*: A case fulfilled with the major symptoms of either ① or ③ and the ancillary testing results comparable for both a) and b). *Possible*: A case fulfilled with either ① or ③ and the ancillary testing results comparable for both b) and c) together with rigorous evidence of a family history of maternal inheritance. *Carrier*: An asymptomatic individual who has one or more maternal relatives either of definite, probable, or probable LHON cases.

### Questionnaire methods

The first questionnaire simply determined the total number and sexes of new patients with LHON during 2014 who satisfied the designated criteria for *definite* or *probable* LHON.^7^ In January 2015, this questionnaire was mailed directly to the heads of 1397 facilities, which comprised 1015 facilities certified by the Japanese Ophthalmological Society, including all university hospitals and 382 facilities with one or more affiliated member of the Japanese Neuro-ophthalmological Society. We asked the heads of these facilities to respond to the questionnaire by the end of March 2015.

After compiling the results of the first questionnaire, we mailed a second questionnaire to the heads of the facilities that reported new patients with LHON in the first questionnaire. The second questionnaire requested detailed clinical and genetic information for individual patients, including information regarding the age of onset and the positions of mtDNA mutations.

### Incidence rate estimation

The number of patients with newly developed LHON during 2014 was estimated using the following formula, in accordance with previous studies^15^, ^16^, ^17^:

$$\;I_{r}=\;\frac{\sum \text{i}\cdot N_{i}}{N/n},$$

where *I*_*r*_ denotes the estimated true number of patients (as units of patients with newly developed LHON), *i* actual number of patients (*i* = 0, 1, 2…), *Ni* number of responding facilities with *i* patients, *N* total number of responding facilities, and *n* the total number of surveyed facilities.

In previous similar epidemiological surveys of intractable diseases,^15^, ^16^, ^17^ the facilities were often selected randomly using stratified sampling from all of the facilities visited by a patient with a disease of interest. Thus, the number of surveyed facilities is usually much smaller than the total number of facilities when the corresponding facilities are classified into a stratum of general hospitals or clinics with a smaller number of hospitalized beds. In this situation, *n* is assumed to be much bigger than the number of facilities surveyed. However, we assumed that incident LHON cases were referred relatively immediately to facilities with affiliated specialists who had knowledge of LHON for the following reasons. First, individuals with a recent onset of LHON notice a rapid and profound decline in their central vision in both eyes, but they rarely exhibit extraocular symptoms. Second, given the necessity for molecular diagnosis and the unresponsiveness to steroid therapy, these patients are highly likely to be referred to tertiary or special institutes with affiliated neuro-ophthalmological specialists. The facilities to which we sent the first questionnaire covered most of these specialists. Thus, all of the facilities were considered to be categorized in the strata of “selected departments” according to previous similar epidemiological surveys of intractable diseases.^15^, ^16^, ^17^ Therefore, we did not consider that the facilities should be stratified based on the hospital type or the number of hospital beds, so *n* was equal to the number of surveyed facilities.

The 95% confidence interval (CI) of *I*_*r*_ was: (*I*_*r*_ − 1.96 × *s*, and *I*_*r*_ + 1.96 × *s*), where *s* is the estimated standard error of *I*_*r*_ computed as follows^15^:

$$\;s=\sqrt{\frac{\sum i^{2}\cdot N_{i}/N-{\left(\sum i\cdot N_{i}/N\right)}^{2}}{n-1}\cdot n^{3}\left(1/N-1/n\right)}$$

A patient reported by multiple facilities was treated as a “duplicate” according to the information obtained from the second questionnaire and/or via direct contact with the physicians.

## Results

We received 861 responses to the first questionnaire from 1397 facilities (response rate, 61.6%), among which 49 facilities reported the presence of newly developed molecularly confirmed LHON cases in 2014. Responses to the second questionnaire were received from 30 of the 49 facilities (response rate, 61.2%).

According to the first questionnaire, there were 72 cases of newly developed LHON during 2014, among which 67 cases were male and 5 were female. No new cases were reported by 812 facilities. Of the 49 facilities that reported new cases, 34 reported one case, 10 reported two cases, 4 reported three cases, and 1 reported six cases. Based on these data, *I*_*r*_ was calculated as 117 (72/[861/1397]) cases in total (109 male cases and 8 female cases), with a 95% CI of 81–153. The proportion of male cases was 93.2%, which was comparable to that found in a previous study by Hotta et al (92.1%).^14^

We confirmed that none of the facilities with more than one case reported any duplicate cases. In addition, among the 34 facilities that reported only one case, 15 confirmed that they had no duplicate cases. We did not confirm this for the remaining 19 facilities (i.e., 19 cases). Therefore, the maximum estimated proportion of duplicates was 26.3%. However, this appears unlikely because each of these facilities was a major referral center in each prefecture and they would have referred cases only to the core facilities that reported more than one case, if any; in fact, all of these major institutes confirmed no duplicates.

**Fig. 1:**
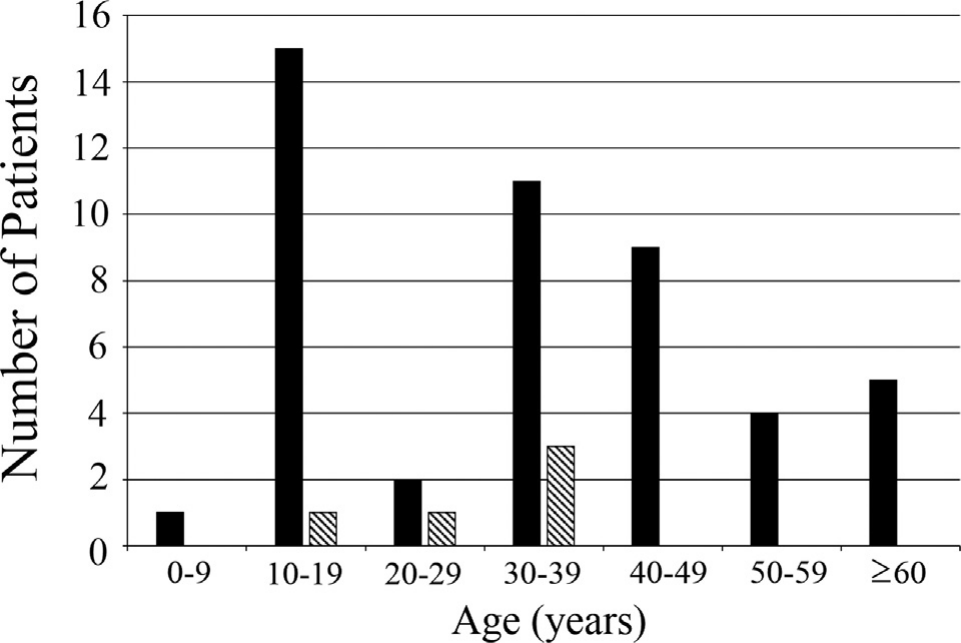
Distribution of age at onset for 44 patients with newly developed Leber hereditary optic neuropathy (LHON) in Japan during 2014. The histogram was generated based on the 30 responses obtained from 49 facilities that reported one or more new patients with LHON in the first questionnaire and that received the second questionnaire. Closed columns indicate male patients and hatched columns indicate female patients.

A composite histogram of the distribution by gender and age at onset for 44 cases based on the 30 responses to the second questionnaire is shown in Fig. 1. The youngest age of onset was 6 years and the oldest was 75 years, where the mean and median ages of onset were 33.5 and 37.5 years (interquartile range, 16–44 years), respectively. The median age for males and females was 38 and 30 years, respectively.

Among the 44 cases reported in the second questionnaire, 38 cases had the ND4/G11778A mutation, 1 had the ND1/G3460A mutation, and 5 had the ND6/T14484C mutation. Thus, 86.4% cases had the ND4/G11778A mutation in this survey, which is similar to the results obtained in previous studies.^14^, ^18^

## Discussion

This is the first nationwide survey to estimate the annual incidence of LHON cases with molecular confirmation in Japan, and we found that *I*_*r*_ for those with newly developed LHON in 2014 was 117 (95% CI, 81–153). Given that approximately 5–10% of patients with LHON have been reported to harbor mtDNA mutations other than the three primary mutations targeted in the current study, it is assumed that the true incidence is higher than this estimated value.

The response rate (61.6%) in this study was better than that in previous evaluations of mitochondrial myopathy, encephalopathy, lactic acidosis, and stroke-like episode (MELAS) (47% for the first questionnaire and 47.8% for the second questionnaire); adult Still's disease (53.6%); and acute pancreatitis (45.8%)^15−17^; still, some patients may have been missed, thereby underestimating the actual incidence. However, this appears unlikely because the facilities that responded to the questionnaire comprised the core institutes for neuro-ophthalmology, so most newly developed LHON cases were more likely to have been referred to these facilities rather than to other facilities. Therefore, it is reasonable to assume that the imperfect response rate led to an overestimate of the incidence rather than an underestimate.

As reported previously,^18^ the ND4/G11778A mutation was the most frequent (approximately 90%) among the three primary mutations. This frequency was considerably higher than its reported frequency (53–60%) in other populations,^1^, ^4^, ^9^, ^10^ which may reflect the unique genetic background of patients with LHON in Japan.

The high proportion of male patients (male:female ratio, 8:1) is in agreement with that found in a previous study (92.1%) by Hotta et al^14^ and is higher than the male:female ratio reported in other countries (3.4:1 in Finland, 5.4:1 in Netherlands, 3.3:1 in England, 3.7: 1 in Denmark, and 6:1 in Serbia).^1^, ^9^, ^10^, ^11^, ^12^ Interestingly, this proportion is also higher than that (68.1%) reported for Japanese patients with LHON in 1973 by Imachi et al,^13^ indicating that penetrance of female carriers may have decreased over the years and that environmental factors may regulate LHON expression.

The age at onset in the present study (mean age, 33.5 years; median age, 37.5 years) was higher than previously reported values, which ranged from 22 to 33 years,^9^, ^10^, ^11^ even compared with that reported for the same ethnic group 20 years previously.^14^ There are two possible explanations for this difference. First, the entry profiles of the subjects differed among studies. Man et al only recruited subjects under 65 years of age,^9^ whereas we did not set a cut-off age for entry. Second, there has been a low birth rate and increased longevity in Japan during the last two decades, which are likely to have shifted the age at onset for LHON to the older.

A previous study analyzed a large Brazilian LHON pedigree with a uniform genetic background and demonstrated that its penetrance declined over generations, which indicates that *I*_*r*_ is influenced by environmental factors.^19^ Smoking is known to be a risk factor for the development of LHON.^8^, ^19^ According to a report by the Ministry of Health, Labour and Welfare, which was based on a survey conducted by Japan Tobacco Inc., the number of smokers in 1966 (83.7% in males and 18.0% in females) had almost halved by 2014 (30.3% in males and 9.8% in females).^20^ This dramatic reduction in smokers may have partly affected the penetrance and the shift in the age at onset of LHON in Japan over the years.

Our approach for *I*_*r*_ estimation has some limitations. The survey-period of 1 year may have been insufficient to capture the total number of newly-developed patients. In addition, it is possible that some cases were overlooked because of unawareness of symptoms, misdiagnosis, and the atypical clinical course. These factors may have led to underestimation of the actual incidence.

In conclusion, this is the first nationwide survey of the annual incidence of LHON in Japan. During 2014, approximately 120 individuals with one of the three primary mtDNA mutations were estimated to have newly developed LHON.

## Conflicts of interest

None declared.
